# Cross-border seasonal migrant labour and agricultural commodity production in the Ethiopia–Sudan borderlands

**DOI:** 10.1007/s10460-025-10716-1

**Published:** 2025-02-26

**Authors:** Tsegaye Moreda

**Affiliations:** International Institute of Social Studies (ISS), Erasmus University Rotterdam, The Hague, The Netherlands

**Keywords:** Commodity production, Cross-border seasonal migrant labour, Social reproduction, Exploitation, Sudan, Ethiopia

## Abstract

The Ethiopia-Sudan borderlands have long been a hub of capitalist production of lucrative agricultural commodities such as sesame that profoundly shape the political economy of these borderlands. Cross-border seasonal migrant labour is central to this capitalist commodity production but is often neglected in discussions, which chiefly focus on economic and geopolitical wranglings between different groups over the control of land and commodity production in these areas. If there is any talk of migrant labour, it is reduced a priori to how these agricultural commodity production sites are crucial in providing employment opportunities to migrant workers and how the flow of such labour should be governed. Thus, the role of migrant labour is rarely recognized, and the associated dynamics of exploitation in its varied manifestations remain obscure. This paper argues that the history and profitability of commodity production in the borderlands are connected to, and rooted in, the availability of vast, cheap, flexible and exploited migrant agricultural labour. This labour is the result of various processes, including land grabs, landlessness, conflict and climate change. The paper argues that the dynamics of exploitation occur not only to seasonal migrants in the capitalist production sites but also to their families and communities back home who shoulder the responsibilities of family subsistence and reproducing the labour of the migrant. The paper contributes to an understanding of the relationship between migrant labour and capitalist commodity production, particularly the crucial role of seasonal migrant labour and the dynamics of exploitation, both in the places of origin and the areas of seasonal migrant work. This will, in turn, contribute to identifying the needs of seasonal migrants and the challenges they face, necessary for informing political struggles for labour justice and socio-economic policies.

## Introduction

How does capitalist commodity production in the Ethiopia-Sudan borderlands shape, and is shaped by, cross-border seasonal labour migration? This paper argues that the history and profitability of commodity production in the borderlands are connected to, and rooted in, the availability of vast, cheap, flexible and exploitable migrant agricultural labour, often the result of various processes including land grabs, landlessness, conflict and climate change. Land and labour are two of the most important productive forces in capitalism and thus are closely intertwined. But the role of labour is now being questioned as it is pushed “from the centre to margins” in the context of a globalized world economy governed by the logic of capital. In recent decades, labour questions have undergone dramatic changes: as Sehgal (2005: p. 2286) points out, “There is no disputing the fact that labour is under attack”, particularly since “the conditions of reproduction of labour are coming under severe strain”, with increasingly informalized and casualized work arrangements that have emerged under neoliberal globalization. The trend has been “to delink the growth of production from that of employment” (Davis 2004: p. 9). The trajectory of global capitalist development, especially over the last two decades, has increasingly drawn land into the dynamics of capital accumulation, while the accompanying processes of dispossession and displacement have created a surplus population whose “land is needed but their labour is not” (Li 2011: p. 286), suggesting that labour may have become less relevant to land-based capital accumulation. This trend needs to be situated in the current broader political economic conjuncture and social change taking place globally.

Large-scale labour migration is “one of the most significant social transformations underway in rural parts of Africa, Asia and Latin America” (Sugden 2019: p. 1600). This is intricately connected to the processes of capitalist accumulation, predominantly characterized by the appropriation and enclosure of land, including common property resources, for capitalist investment across the global South, driving rural people away from land towards labour migration (Borras and Franco 2012; White et al. 2012; Hall et al. 2015; Nyantakyi-Frimpong & Bezner-Kerr 2017). The convergence and interaction of multiple processes such as global food security concerns, energy and financial crises, urbanization, increased global demand for commodities, climate change mitigation imperatives, etc., have resulted in land and landed resources regaining prominence as critical resources in the 21st century (Borras and Franco 2012). As a result, different scales of land and resource grabbing, taking place in various ways and involving a variety of actors, have surged across much of the global South but also in many countries in the global North (Neef et al. 2023). While land enclosures and grabs are not necessarily new, “[w]hat is new in the land grabs today are the new mechanisms of land control, their justifications and alliances for ‘taking back’ the land, as well as the political economic context of neoliberalism that dominates this particular stage of the capitalist world system” (Peluso and Lund 2011: p. 672).

One of the key issues in the global land grabs literature is the implication of land deals for labour regimes and labour processes, focusing on how changes in the access, use and control of land shapes labour dynamics (Jha et al. 2021.; Gyapong 2020; Ouma 2018; Nyantakyi-Frimpong & Bezner-Kerr 2017; Hall et al. 2017; Oya 2012; White et al. 2012; Li 2011). With land grabs, new labour regimes – understood here as “the specific methods of mobilizing labour and organizing it in production, and their particular social, economic and political conditions” (Bernstein 1988: pp. 31–32) – emerge and result in the restructuring of not only who works on the land, under what arrangements and with what implications for differently positioned social groups but may also shape patterns of migration. There is already a vast literature on the kind of labour regimes associated with large-scale capitalist agriculture. Capitalist farming models – currently being widely promoted – which involve a shift from individual and communal to corporate control of land, are often labour-expelling and do not create enough jobs (Hall et al. 2015; Borras and Franco 2012; White et al. 2012; Li 2011). Those that are created are often casual and seasonal (Gyapong 2020; Ouma 2018; Nyantakyi-Frimpong and Bezner Kerr 2017; Hall et al. 2015) and of low-quality (Gibbon 2011).

Through land enclosures and dispossessions, populations are turned into relative surplus due to the limited labour requirements by capital-intensive investment ventures in farming (White et al. 2012; Li 2011). Such capitalist ventures thus create an “agrarian question of labour” (Bernstein 2004). While a limited absorption of labour is one of the key features of capital-intensive farming, capital accumulation in agriculture can also seize labour through distinct capitalist labour processes such as contract farming, through which smallholder farmers become labourers on their own land, or ‘propertied labourers’ (Oya 2012; McMichael 2013; Little and Watts 1994). That is, smallholders can be proletarianized without being directly dispossessed of their land. It means that existing specific land uses can change, and land can be drawn into the accumulation dynamic without a change in ownership. Under such labour processes, the autonomy of peasants is undermined and their labour disciplined (Little and Watts 1994).

In the current phase of neoliberal capitalism, a significant proportion of the world population is surplus to the requirements of capital accumulation (Sugden 2019; White et al. 2012). As Marx (1976: p. 782) articulated, “in fact it is capitalist accumulation itself that constantly produces… a relatively redundant working population, i.e. a population which is superfluous to capital’s average requirements for its own valorization, and is therefore a surplus population”. As elaborated by White (2011: p. 4), “a historically-specific relative surplus population, itself the result of capital accumulation and technical progress which is surplus not to society’s capacity to provide subsistence but to capital’s requirements for labour, resulting in low wages of the employed and pauperism of the un- and underemployed even in contexts of rapid economic development… It is not only agriculture, but also many other sectors whose ‘development’ through capital investment and technical change involves the shedding, rather than the absorption, of labour” (While 2011: p. 4).

The dynamics and implications of land grabbing for labour have been discussed and debated in the context of the continued importance of access to land for the economic and social reproduction (i.e., the capacity to sustain everyday life/survival) of rural working people (Yeni 2024; Gyapong 2020; Ouma 2018; Mezzadri et al. 2024), even though the relative dependence on land for livelihoods has changed as livelihood sources have become increasingly diversified (Rigg 2006; Bryceson 2002). Growing numbers of people increasingly depend on the sale of their labour power for their daily reproduction (Jha et al. 2021). Bernstein’s notion of ‘classes of labour’ captures those differentiated classes who “pursue their means of reproduction across different sites of the social division of labour: urban and rural, agricultural and non-agricultural, wage employment and self-employment” (Bernstein 2008: p. 251). Breman (1996) talks of ‘footloose labour’, referring to a working population locked in circular migrations; constantly moving between their villages and other places to wherever they can find (often precarious) work. The fragmentation of labour denotes “the effects of how classes of labour in global capitalism, and especially in the South, pursue their reproduction, through insecure and oppressive – and in many places increasingly scarce – wage employment, often combined with a range of likewise precarious small-scale farming and insecure informal-sector (‘survival’) activity, subject to its own forms of differentiation and oppression along intersecting lines of class, gender, generation, caste and ethnicity” (Bernstein 2008: p. 250 − 51). This fragmentation of labour occurs in the spheres extending from the rural economy to the urban economy and is closely related to the increased ‘informality’ of economic activities (Davis 2006). Thus, ‘the informalization of labour’ in both the rural and urban economy, more precisely in the rural-urban continuum, cannot be overlooked (Breman 1996; Davis 2006). In fact, these working people in all sectors of the economy, including those self-employed in the informal sector, are fundamental to capital accumulation under neoliberalism (Fraser 2016). As Shivji (2017) contends, the concept of ‘working people’ refers to producers who super-exploit themselves by engaging in multiple occupations to survive while subsidizing capital. Shivji argues that “the materiality that underlies producers – peasants and pastoralists, proletarians and semi-proletarians, street hawkers selling consumer goods and peddlers selling cooked food, operators and repairers in backyard workshops – in virtually all sectors is the minimizing of their necessary consumption and maximizing their labour” (Shivji 2017: p. 11). In rural settings this might, for example, mean that farmers who have access to small plots of land are inserted into contract farming arrangements where they are made to intensify their commodity production using their own and their household’s labour intensively and work simultaneously as wage labourers while reducing their level of consumption, which Bernstein might frame as a “simple reproduction squeeze”. Due to current land grabbing, which often not only dispossesses current land users and changes existing land uses but also fails to provide adequate employment, rural working people increasingly engage in pluri-active livelihood activities in all sectors of the economy. Particularly, the growing numbers of “informal workers have to work extremely hard to be able to eat” (Bernstein 2007: p. 5).

Such dynamics of informality are a key feature of employment worldwide, and a significant number of the global workforce remains in informal and vulnerable employment: latest estimates show that people informally employed account for 58% of global employment (ILO 2024: p. 15). The number of workers in informal jobs reached 2.03 billion in 2024, up from 1.83 billion in 2015 (1.7 billion in 2005), while the number of people without a job but wanting to work is estimated at 402 million (ILO 2024a: p. 10, 4). This scale of informality raises concerns about overall working conditions and precarity, including job insecurity, lower wages, exploitative working conditions and lack of access to collective bargaining since many of the workers in informal employment lack adequate social and legal protections (Mezzadri 2021; Fraser 2016). The exploitation of labour is a mark of informal employment which is characterized by “the absence of formal contracts, rights, regulations, and bargaining power. Petty exploitation (endlessly franchised) is its essence” (Davis 2006: p. 181; see also Breman 2003: p. 196). As Davis puts it, the informal sector is “a living museum of human exploitation” (Davis 2006: p. 186). Strikingly, the scale of informality is particularly significant among the youth. For instance, 96.8% of young people in developing countries work in the informal sector (UN 2020: p. 45). “As a result, in the daily struggle to survive, this [flexibilized working population] is condemned to perpetual mobility in the search for work, both within and between sectors and between modes of employment” (Breman 2003: p. 231) and between and across (sub)national borders.

Seasonal migrant workers are “increasingly associated with the informalisation and casualisation of employment” (Cousins et al. 2018: p. 1081; see also; Breman 2003). Seasonal migrant workers usually work in the informal economy. And even when they work in the formal economy, it is often through informalized employment characterized by casual contracts, poor living and working conditions, low wages and the lack of access to basic workers’ rights – a significant feature of and crucial to the exploitation of seasonal migrant labour (Borras et al. 2022). In this paper, these seasonal migrant farmworkers are therefore understood as the sections of the working class that are captured by relevant concepts such as ‘working people’ (Shivji 2017), ‘fragmented classes of labour’ (Bernstein 2008) and ‘footloose labour’ (Breman 1996).

## Dynamic entanglement of commodity production, social reproduction and labour exploitation

Deploying the concept of social reproduction from a Marxist feminist perspective is essential to understanding everyday life under capitalism and hence the dynamics of seasonal migrant labour exploitation. Social reproduction refers to “the complex of activities and relations by which our life and labor are daily reconstituted” (Federici 2012: p. 5). It comprises the “the material and discursive practices which enable the reproduction of a social formation (including the relations between social groups) and its members over time” (Wells 2009: p. 78). Of particular relevance to this paper is that “human labour is at the centre of the reproduction of households, communities and societies, and that this labour includes not only the production of marketable goods and services but also the reproduction and maintenance of life itself – sometimes called ‘reproductive work’ – as parts of a single, integrated process” (White 2020: pp. 15–16; Bhattacharya 2017). Increasingly, Marxist feminists see social reproduction as an integral component of capitalism and its longstanding entanglement with gendered labour. They conceptualize the processes of the production of commodities and the reproduction of labour power in households, communities and the niches of everyday life as being unified instead of seeing them as belonging to separate processes (Bhattacharya 2017; Fraser 2016; Mezzadri 2021). The spaces of production and reproduction “may be separate in a strictly spatial sense, but they are actually united in the theoretical and operational senses” (Bhattacharya 2017: p. 74). It may sometimes be the case that production and reproduction take place within the same space.

As Federici (2004; 2012) argues, capitalist accumulation was historically contingent on the sexual division of labour that confined women to labour-intensive reproductive housework and the exploitation of their labour. Within households, most socially reproductive work is largely dependent on women’s unpaid labour (Yeni 2024; Naidu and Ossome 2016; Ossome 2021; Tsikata 2016; Benya 2015; Razavi 2009). In rural areas of the global South, the activities performed by women that underlie daily reproduction include preparing meals, fetching water and firewood, cleaning and caring for children, the elderly and sick family members while simultaneously engaging in ‘productive’ work such as subsistence agriculture and other livelihood activities (Yeni 2024). The fact that the provision of sanitation, piped water and electricity are generally limited in many contexts in the Global South means that women spend a considerable amount of time and energy on such domestic and care work (Yeni 2024). Therefore, commodity production that is fundamentally premised on the sale of cheap seasonal migrant labour is “reproduced on a daily basis by the invisible [or taken-for-granted] labour of countless women” (Benya 2015: p. 546). Nevertheless, although social reproductive work relies heavily on women’s work, it involves kinship/family and community networks (Shah and Lerche 2020) as well as institutions, processes and social relations associated with creating and maintaining households and communities (Bakker and Silvey 2008: p. 2). This socially reproductive work “overwhelmingly, although not exclusively performed by women” (Ossome 2021: p. 560), is crucial to the exploitation of seasonal migrant labour.

Interestingly, the realm of exploitation of migrant labour is not merely linked to conditions at the commodity-production sites where migrants work but also linked to the migrants’ families and villages back home (Mezzadri 2021; Shah and Lerch 2020; Cousins et al. 2018; Sugden 2019). Capitalist production is based on the extraction of labour-surplus that occurs through multiple forms of exploitation (Banaji 2010). In this regard, wage-labour is among the many forms in which exploitation may take place. Exploitation may also be linked to non-waged spheres of production and social reproduction. Social reproductive activities and realms contribute to the extraction of labour-surplus through, for instance, the processes of circular labour migration when employers can dump the cost of family subsistence and labour reproduction onto workers’ households, families and communities in the migrants’ place of origin (Mezzadri 2021; Cousins et al. 2018; Benya 2015). This suggests the importance of considering the processes and relations through which households, families and communities are constituted in such dynamics (Cousins et al. 2018: p. 1082). Therefore, to understand the dynamics of exploitation of seasonal migrant labour, recognizing the relation between productive and reproductive activities and realms as one of co-constitution is crucial (Mezzadri 2021; Shah and Lerche 2020). Mezzadri (2021: p. 1186) argued, this requires “stressing the dynamic interpenetration of production and reproduction in processes of labour-surplus extraction”. Most seasonal labour migrants in the global South are “rurally rooted” meaning that “they have not completely cut ties to their rural roots”, which also means that maintaining access to land in their place of origin remains critically important for social reproduction (Borras et al. 2022: p. 317; see also Cousins et al. 2018; Yeni 2024). This rural rootedness and continued access to land are crucial for covering the cost of family subsistence and labour reproduction as the migrants do not usually earn much from their migrant work. Partly because of this, “[t]he use of seasonal migrant labour is particularly profitable for employers because they can be exploited more than local labour in multiple spaces: the receiving areas where migrants work and live, as well as home regions which are crucial for migrant social reproduction” (Shah and Lerche 2020: p. 726). In the same vein, in highlighting the history of the development of South Africa’s capitalist economy, Cousins et al. note that “self-provisioning through small-scale farming for domestic consumption and sale underpinned a system of cheap, migrant labour” (2018, pp.1061). Similarly, Benya (2015) shows how the unpaid reproductive work performed by women not only enabled men to work in the mines but also to sell their labour for low wages.

Labour migration within Ethiopia and to the border areas of eastern Sudan to seek seasonal agricultural wage employment on large commercial farms has been a recurring phenomenon since at least the 1960s. While this seasonal migration currently constitutes mostly landless young male rural labourers, it also includes male household heads who continue to have access to small plots of land in their villages. However, because their land is deemed insufficient for their social reproduction, this latter group also engages in circular migration as a necessary complement to what they can earn on their small plots. Commodity production in Ethiopia in the form of industrial/export commodities such as cotton, tobacco, sugarcane, sesame and coffee, dates back to the same period. However, with the onset of the recent global land rush, Ethiopia quickly became a global hotspot attracting foreign direct investments in its farmland. Even before the recent global rush for farmland, large-scale export-oriented agriculture had already been a central pillar of Ethiopia’s development policy (Rahmato 2014). It is in this historical context that commercial agricultural areas have been seen as crucial in providing seasonal employment opportunities for hundreds of thousands of migrant workers. What is overlooked, however, is how the availability of vast, cheap and exploitable migrant labour has enabled the expansion of commercial farms. This relates closely to understanding the location of labour in the process of capitalist accumulation in that “if a surplus population of workers is a necessary product of accumulation or of the development of wealth on a capitalist basis, this surplus population also becomes, conversely, the lever of capitalist accumulation, indeed it becomes a condition for the existence of the capitalist mode of production” (Marx 1976: p. 784).

The lowlands between northwestern Ethiopia and eastern Sudan have long been a hub of capitalist production of agricultural commodities. The production of high-value crops such as sesame and cotton profoundly shape the political economy of these borderlands. The availability of vast fertile land watered by several rivers is central to the production of agricultural commodities, making these areas economically very important for both countries. Over half a million Ethiopian seasonal agricultural labourers, predominantly from the Amhara and Tigray regions, move across the lowlands between Ethiopia and Sudan to work on farms on both sides of the border (ILO 2020a: p. 14; Eldin and Ferede 2018). These seasonal migrant farmworkers predominantly are men. On the Ethiopian side, labourers work on farms in Metema and Humera; across the border they work on farms in eastern Sudan, especially around Gedaref state. Such cross-border seasonal migrant labour forms an important factor in the capitalist commodity production in these lowland borderlands but is often neglected in discussions, which chiefly focus on economic and geopolitical wrangling between different groups over the control of land and commodity production in these areas. If there is any talk of seasonal migrant farmworkers, it is reduced a priori to how these agricultural commodity production sites are crucial in providing employment opportunities for migrant workers and how the flow of such labour should be governed. Therefore, the role of seasonal migrant labour is rarely recognized, and the associated dynamics of exploitation in its varied manifestations remain obscure. This paper argues that the history and profitability of the capitalist production of commodities in the Ethiopia-Sudan borderlands are connected to, and rooted in, the availability of vast, cheap, flexible and exploited cross-border migrant agricultural labour, often produced by different processes including land grabs, landlessness, food insecurity, conflict and climate change. The dynamics of exploitation of these seasonal migrant farmworkers occur and manifest in varied forms and across multiple sites.

Shah and Lerche (2020) argued that the intimate relationship between production and social reproduction is crucial to seasonal migrant labour exploitation. Informed by Shah and Lerche’s articulation and set in the context of commercial farming areas in the Ethiopia-Sudan borderlands, this paper argues that the dynamics of exploitation occur not only to seasonal migrants at commodity production sites in the borderlands but also when their households, families and communities back home shoulder the household subsistence and labour reproduction activities that enable the labourers to migrate. This paper particularly focuses on the latter domain, looking to understand the relationship between migrant labour and the dynamics in their villages of origin. The paper draws from data collected in the Amhara region, particularly the Tach Gayint district, one of the region’s major seasonal migrant-sending districts to commercial farms. Through extended fieldwork, I conducted key informant interviews and focus group discussions with seasonal migrants and their families and conducted a survey with 300 randomly selected households (Moreda 2016). Tach Gayint is a food-insecure district where most households are food-deficit and rely partly on support through cash- and food-for-work programmes. For the district’s households, most of which are confronted not only with a shortage of land and environmental degradation but also with problems of access to other means of subsistence, seasonal labour migration to the northwestern lowland borderlands is crucial for their livelihoods. Of 300 sampled households in Tach Gayint, 186 (62%) had at least one member who migrated for employment in the 12 months preceding the survey. Yet only seven of the 300 households surveyed did not have access to land. Most owned a certain amount of land under the existing usufruct land tenure system, although plot sizes were generally small and of poor soil quality due to widespread land degradation.

The remainder of the paper is organized as follows. The next section introduces the Ethiopia-Sudan borderlands, where commercial agriculture is already well-entrenched and attracts hundreds of thousands of seasonal migrant farmworkers. This is followed by an overview of the dynamics and trajectory of commodity production in Ethiopia. The fourth section discusses the pattern of seasonal migrant labour and the dynamics of exploitation surrounding it. The final section presents a short conclusion.

### The borderlands

Ethiopia and Sudan share a 740 km land border, part of which the two countries have contested for over a century, with the exact boundary is rarely demarcated on the ground. The initial attempt to demarcate the boundary was established by the Anglo-Ethiopian Treaty in 1902 (when Sudan was still under British rule), followed by a unilateral effort by British surveyors to demarcate the boundary in 1903. However, Ethiopia never accepted this unilateral demarcation effort. In 1972, Ethiopia and post-independence Sudan sought a negotiated re-demarcation of the borderline through the Exchange of Notes (Soliman and Demissie 2024). Despite subsequent efforts, the boundary between the two countries remains unresolved. The two countries have long competed for control of the highly valued land and agricultural production in the border areas, with localized territorial conflicts a common occurrence. Various actors (both state and non-state) within and between the two countries have competed to control the fertile land and the highly lucrative sesame production in these borderlands, making them hotspots of large-scale, exported-oriented, partially mechanized commercial agricultural production that depend on a huge agricultural workforce. Commodity production by smallholders in the area is also significant, particularly the widespread practice of claiming land deemed unoccupied and farming it temporarily on a seasonal basis (Gezahegne 2020; REF 2016c). Many communities living in these borderlands share cross-border economic, linguistic, cultural and ethnic ties, with cross-border farming, livestock herding and trade constituting their main sources of income and livelihood. In addition, seasonal agricultural work, from both sides of the border, has long been a major source of income. Sudanese farmers usually rely on migrant farm workers crossing from Ethiopia through legal and illegal channels during planting and harvesting seasons. Ethiopian migrant farm workers move back and forth across the border between Sudanese and Ethiopian commercial farms. However, the exploitation of these workers by various actors, including commercial farmers, brokers and border officials, is widespread (Gallopin et al. 2021; Gezahegne 2020; REF 2020).

**Map 1:**
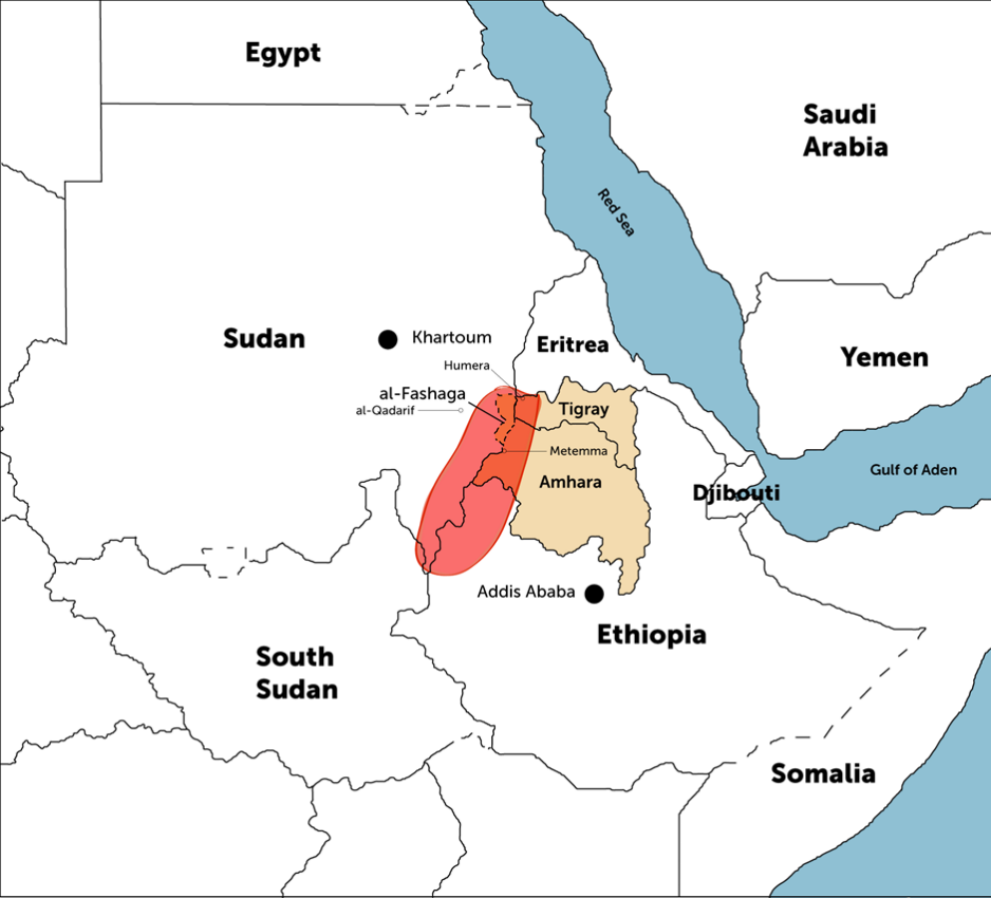
The Ethiopia-Sudan borderlands (shaded in red). Source: Adapted from ‘Ethiopia-Sudan border tensions must be de-escalated’ by Woldemichael (2021), Institute of Security Studies. https://issafrica.org/iss-today/ethiopia-sudan-border-tensions-must-be-de-escalated.

One of the historically contested areas where border farming takes place and serves as a site of employment that draws hundreds of thousands of seasonal farm workers is the area known as the Al-Fashaga triangle: an area of about 243,000 hectares of vast fertile agricultural land watered by three rivers – Tekeze, Atbara, Angereb (Soliman and Demissie 2024). This agricultural heartland is claimed by both Sudan and Ethiopia, with farmers from Sudan’s Gedaref state and from the Amhara and Tigray regional states in Ethiopia competing to access farming and cattle-raising land. Due to these competing claims, “lands claimed by Ethiopians in one farming season might be claimed and farmed by Sudanese during another season” (Eyilet and Senishaw 2020: p. 15). These borderlands are areas where major, labour-intensive export crops such as sesame and cotton are cultivated. Stretching from where north-west Ethiopia meets Sudan’s breadbasket Gedaref state, these borderlands extend further south, with the Benishangul-Gumuz regional state of Ethiopia sharing a border with Sudan’s Blue Nile state. On the Ethiopian side of the border, the major hubs of large commercial farms attracting a large migrant workforce include Metema, Humera and Metekel. The major hubs of commercial farming schemes in the eastern borderlands of Sudan include Gedaref, Kassala, Blue Nile and Sennar states. These borderlands are marked by widespread cross-border mobility and bustling border towns where diverse groups, including border communities, traders, farmers, pastoralists, seasonal farm workers, brokers, human smugglers and traffickers, sex workers, state authorities, militias and bandits, rebels and so on interact. The two-year catastrophic civil war in the Tigray region, which ended in a peace agreement in November 2022, the ongoing violent conflict in the Amhara region and the raging war in Sudan that erupted in 2023 have significantly impacted border communities, particularly by creating challenging environments for seasonal farmworkers, even exposing them to further exploitation by various interest groups. These borderlands have now become increasingly militarized.

## The dynamics and trajectories of commodity production and seasonal migrant labour in Ethiopia

Historically and at present, the extent of agrarian proletarianization in Ethiopia has been limited. Agrarian proletarians are understood as classes of labour dependent on agricultural wages for their social reproduction. The major areas in Ethiopia that have historically attracted a large number of agricultural proletarians or farm workers had been limited to the Awash Valley (which attracted investments for irrigated commercial agriculture and agro-industries), the rainfed commercial farms in the northwestern lowlands and the coffee growing areas in the southwestern part of the country (Rahmato 2009). From the early 1960s, large-scale commercial farms started flourishing in the Awash Valley as a response to changes in government policy that emphasized import substitution and an increase in exports. Particularly under Ethiopia’s ‘Third Five-Year Development Plan’ (1968–1973), the government stressed large-scale commercial agriculture as a way of developing agrarian capitalism (Alemu 2002).

In the 1960s, foreign companies established commercial farms growing mainly sugarcane and cotton on a large scale on irrigated lands in the Awash Valley. In fact, some farms had been established even earlier, in the 1950s (Bondestam 1974; Markakis 2011). For instance, on large tracts of land acquired through long-term lease arrangements, the Dutch joint venture company HVA-Ethiopia established the first sugarcane plantation at Wonji that started commercial production of sugar in 1954. The company established more sugar plantations at Metahara in the late 1960s. By 1970-71, over 25% of an estimated 200,000 hectares of irrigable land in the Awash Valley had already been brought under commercial cotton and sugar production (Bondestam 1974: p. 428; Markakis 2011: p. 138). However, large commercial farming enterprises were not the only ones developing the valley at the time, with enterprising small farmers also playing a significant role (Rahmato 2006: p. 11). Following the opening of the Awash Valley for commercial development, many small farmers from the adjoining highlands migrated to the area. Many of them rented irrigated land where they produced crops such as cotton and sugarcane and supplied their produce to the large plantations and agro-industries via contract farming/outgrower arrangements (Emmanuel 1975: p. 15). It is estimated that some 20,000 outgrowers were operating in the valley at the beginning of the 1970s, cultivating about one-third of the valley’s total irrigated land (Rahmato: 2006: p. 11; 2009: p. 89). Some pastoralists from the valley also adopted settled farming and became cultivators producing cotton on the irrigated plains (Kloos 1982a, b; Emmanuel 1975).

Before the development of capitalist irrigated agriculture in the area, the Awash Valley had been almost entirely used by pastoralist tribes for livestock grazing and some crop farming on the flood plains (Emmanuel 1975; Markakis 2011). The growth of commercial agriculture resulted in evictions of pastoralists from parts of the valley (Emmanuel 1975; Markakis 2011) with some estimates indicating that around 20,000 pastoralists were displaced by agricultural development schemes (Kloos 1982a: p. 29, 1982b: p. 154). As historian Bahru Zewde noted, “The inhabitants of the valley were shunted off to less fertile areas. Hanging on to the slopes of nearby hills and eking out a miserable existence, they looked on as sugar and cotton (by Dutch and British capital, respectively) dominated the scene” (2008: p. 121).

The private commercial farms that had expanded under the imperial regime in the Awash Valley and other parts of the country were nationalized after the 1975 land reform. The plantations were later converted into state farms, and some were distributed to the surrounding peasants (Markakis 2011; Rahmato 2009). Rahmato’s estimate puts the total land size under mechanized commercial farming in the country before the land reform at 320,000 hectares (2009: p. 84). It should be noted here that a ban on the hiring of agricultural wage labour introduced after the land reform had greatly affected the development of the agricultural proletariat in the country (Rahmato 2009). Indeed, by the end of Haile Selassie’s regime in 1974, the commercial agriculture sector in Ethiopia, as Rahmato wrote, “had evolved to a stage where a form of agrarian capitalism was emerging as a force in the countryside” (2009: p. 81), but that emerging force was prematurely brought to a halt by the land reform (ibid.: p. 97).

Alongside the Awash Valley, Humera and Metema in the northwestern lowlands, where rainfed commercial crops such as sesame, cotton and sorghum have thrived since the early 1960s, have attracted tens of thousands of seasonal migrant farmworkers (Rahmato 2009). Likewise, the coffee-growing regions in the southwest, the cereals-producing areas in Arssi in the southeast and the commercial farms in the Rift valley have also been destinations attracting a considerable number of migrant agricultural workers (Negash 2017; Kloos 1982a, b). From the beginning, these seasonal workers were mostly migrant wage labourers originating from areas outside where the commercial farms were located. For instance, the seasonal plantation workers in the Awash Valley included those that had been recruited annually through the plantations’ recruitment programs in some significant migrant labour source areas such as Kembata and Hadya, and those who ventured on their own to the commercial farms (Kloos 1982a, b). Without accounting for the farmworkers in the lower plains of the Awash Valley, Kloos ([Bibr CR51][Bibr CR50]: p. 154) estimates that there were 27,000 seasonal and daily labourers in the upper and middle Awash Valley schemes in 1975/76. As will be discussed later, to this day, the northeastern and northwestern lowlands of Ethiopia continue to be major hotspots for hundreds of thousands of migrant farmworkers from various parts of the country seeking employment every year, despite setbacks to commercial agriculture during the Derg regime (Alemu 2002; Rahmato 2009).

## Contract farming and labour

Discussions surrounding agrarian labour regimes and processes and capitalist development in agriculture are not limited to plantations but also relate to outgrower schemes. Sugarcane outgrower schemes have been expanding in the Awash Valley since 2008 (Wendimu et al. 2016), although the oldest such scheme was established in the early 1970s (Emmanuel 1975). The expanding number of outgrowers in the area supply the sugarcane they produce exclusively to the Wonji-Shoa Sugar Factory. The participation of smallholder farmers in the outgrower schemes was, in a way, involuntary as the farmers holding land adjacent to the Wonji-Shoa sugarcane plantations were compelled to either participate or leave their land without compensation (Wendimu et al. 2016). This is, of course, a reminder of the problems inherent with the notion of contracts in outgrower schemes as being agreements willingly made between equal parties. Such production relations are actually shaped by and embedded within the terms and conditions governing access to land and existing power relations. Capturing Kautsky’s proposition of the multilinearity of the paths of capitalist development in agriculture, Watts states that “large landholding no more sounds the death knell for the small allotment holder than agrarian capitalism demands the free landless worker” (1989: pp. 29–30). Part of the reason for smallholders’ ability to reproduce themselves in areas where the agricultural sector is penetrated by capitalism is that they were deemed important and hence not expropriated, i.e., they were the source of cheap labour required by large-scale capitalist farmers. Under contract farming, smallholder farmers are converted into a labour force working on their own land under contract relationships. The assertion is that “capitalism can penetrate agriculture and transform existing social relations in a variety of ways that result in different class and land tenure configurations” (de Janvry 1981: p. 106).

It is essential to go beyond the notions of undifferentiated outgrowers towards understanding the class structure within outgrower schemes (Niño 2016; Oya 2012; White 1997). While considerable attention has been given to contract arrangements as a way of linking smallholders with agribusiness, research about the nature and relations of outgrowers and those who work for them, such as wage labourers and sharecroppers, has been limited. While outgrowers are generally assumed to be smallholders cultivating their own land using family labour, this may not always be the case (Hall et al. 2017: p. 519). They may actually be small-scale farmers using hired labour (White 1997) or rented land. Furthermore, increased labour demands for contract production may be internalized within domestic (intrahousehold) relations of production and reproduction, often producing tensions and competing interests over access to labour and property (O’Laughlin 2008; Little and Watts 1994; Kirk 1987). This is the case partly because, in contract farming arrangements, the farmer agrees “to sell his *crop*, not his labour” (Kirk 1987: p. 47), since such arrangements leave production in the hands of contracted farmers. This means all household members may be labouring long hours to meet the expected quantity and quality of the crops specified in the contract, underscoring the Chayanovian notion of self-exploitation of the peasant household (or perhaps what Kautsky calls self-exploitation: cheap self-exploited household labour as the basis of peasant competitiveness). Consequently, labour processes (including exploitations) and associated conflicts are internalized within the household. It is essential to see “households as domestic structures, configurations of social processes and forms of commodity production under capitalism” (Watts 1989: p. 13). This underscores the importance of focusing on the rural labour process as “a vehicle to examine the way in which local (including domestic) institutional rules and struggles shape, and yet are produced and constrained by, larger political-economic structures of which they are part” (Watts 1989: p. 19).

## Implications of large-scale land acquisitions for labour

In recent years, there has been a growing interest in critically analysing how (trans)national land deals may have altered agrarian structures and spurred displacement, dispossession and proletarianization. In this regard, the leasing of large tracts of land in Ethiopia, which accelerated significantly from 2008, prompted a revival of old debates from the 1960s and early 1970s on the development of the large-scale commercial agriculture sector. As in the past, the focus now is on understanding the role of current large-scale land deals in pushing new cycles of displacement and dispossession of peasants and pastoralists on the one hand and in promoting national ‘growth and transformation’ on the other.

It should be noted that unemployment and underemployment are currently high in Ethiopia (NPC 2016), but this is not entirely attributable to expulsion from land. Nevertheless, an increasing number of people have been dispossessed, and the threat is enormous (Rahmato 2014). As the non-agricultural sector cannot yet adequately absorb surplus labour, while land is being increasingly drawn into the global capital accumulation dynamic, the implications of recent land acquisitions for labour are profound and underscore the continued relevance of the land and labour. The fact that the agricultural models being promoted are capital-intensive ventures that expel peasants and (agro-) pastoralists from the land means the promise of employment creation at a significant scale has not and cannot be real (Rahmato 2014; Shete and Rutten 2015). Nowadays, as White writes, “it is not only agriculture, but also many other sectors whose ‘development’ through capital investment and technical change, involves the shedding, rather than the absorption, of labour” (White 2011: p. 4).

It is important to place the question of land and labour in the broader context of socio-economic and political transformations taking place in Ethiopia. While Ethiopia has achieved rapid economic growth and the unemployment rate has declined in both rural and urban areas over the last decade, unemployment, which was estimated at 8% in 2021, continues to pose a key challenge, particularly for the youth (CSA 2021: p. 9; NPC 2016; World Bank 2021). The country remains one of the least urbanized in sub-Saharan Africa: 79% of the country’s population continues to live and work in rural areas (UNPFA 2019; AfDB 2019: p. 52). Access to land thus remains critical.

Although the share of agriculture in Ethiopia’s GDP has declined over the past two decades, it remains significant. Between 2003/04 and 2022/23, the share of agriculture in GDP declined from 55 to 32%, whereas industry’s share increased from 13 to 28% and that of the services sector increased from 33 to 40% (AfDB 2024: p. 205; World Bank 2019: p. 9). Despite this, agriculture remains the most dominant source of employment, accounting for 63% of all employment in 2022 (World Bank 2024). Ethiopia currently has a rapidly growing labour force, and the high rate of urban unemployment, especially among the youth, is aggravated by high rural-urban migration. The 2021 national labour force survey shows that rural-urban migration was 32.2%, while rural-rural migration accounted for 23.4% (CSA 2021: p. 14).

Part of the reason for the high rural-urban migration is that the rural youth are facing increasing difficulty in accessing land and risk becoming landless (Yeboah et al. 2019; Kosec et al. 2017; Schmidt and Bekele 2016; Bezu and Holden 2014; Headey et al. 2014). Of course, not having access to land may not necessarily represent a dead-end for rural youth if they can be absorbed into other sectors of the economy. But the ability of the non-farm sector to absorb the rural labour force exiting agriculture is limited (Schmidt and Bekele 2016). Even though people migrate from rural to urban areas searching for jobs, unemployment in urban areas is actually almost three times the rate it is in rural areas (World Bank 2021: p. 14). Strikingly, it is estimated that the size of the working-age population in Ethiopia is projected to grow by two million per year over the next 10 years, which will dramatically increase the demand for jobs (World Bank 2019: p. 55).

Ethiopia’s manufacturing sector has been rapidly growing since 2005, but its role in terms of employment generation remains marginal (Oqubay 2019). The total number of jobs in Ethiopia’s manufacturing sector increased from 1.4 million in 2014 to about 1.7 million in 2019 (World Bank 2021: p. 13). Under GTP II, the government emphasized the urgent need to generate sufficient employment for the country’s rapidly expanding labour force, primarily through the development of the manufacturing industry. Consequently, the government embarked on developing about 25 industrial parks across the country. Some of these parks are already operational, creating tens of thousands of manufacturing jobs (Oqubay 2019). However, the government’s efforts recently faced setbacks as the quota and tariff-free access to the US market through the AGOA was suspended, affecting tens of thousands of jobs already created.

It is thus in such contexts that large-scale, state-driven land acquisitions have taken place, involving the displacement and dispossession of peasant cultivators and (agro) pastoralists from their land. The assumption has been that large-scale land acquisitions for commercial farms will create new labour opportunities attracting migrant workers. Yet, as empirical studies have shown, state-facilitated land acquisitions have displaced people in various parts of the country and undermined the ability of local communities to obtain a livelihood from land resources. In areas where displacement has not yet occurred, the threat is clear (Abate 2019; Regassa et al. 2019; Tura 2018; Shete and Rutten 2015; Rahmato 2014) as the government remains determined to transfer more land to investors even though many of the land investments have collapsed or failed to deliver on their promises (NPC 2016). Displacements have not only been from rural lands but also from urban/peri-urban lands. These displacements and the recasting of relations around land and labour contribute to expanding the number of ‘surplus labourers’ whose prospects of accessing land is limited and who seldom find jobs in the non-farm sector.

In addition to land investment-induced displacement, over the last five years Ethiopia has also experienced unprecedented levels of internal displacement, caused by ethnic violence and civil war and climate-induced displacement mainly the result of drought and floods. There were over 4.5 million IDPs across the country at the end of 2022, with conflict and violence accounting for over 3.8 million of these. In 2022 alone, over two million internal displacements due to conflict and violence and about 873,000 due to other disasters were recorded in Ethiopia (IDMC 2023: p. 137). This number is likely to increase due to the ongoing conflict and violence within the Amhara and Oromia regions. Ethiopia is also one of the largest refugee-hosting countries in Africa, hosting 972,835 registered refugees as of February 2024 (UNHCR 2024). Large-scale land acquisition hotspot regions of Gambella and Benishangul-Gumuz are among the principal refugee-hosting areas. Given the large refugee population, Ethiopia has recently taken a much-welcomed approach which provides refugees with work permits. The Ethiopian government has also promised to create employment opportunities for them. This includes reserving 30% of the available jobs for refugees in the new industrial parks being established as part of the country’s industrialization drive (Hammond 2019). Information is limited as to whether this has been realized, given the recent set back that affected manufacturing jobs within the industrial parks due to the suspension of Ethiopia from quota and tariff-free access to the US market. Ethiopia’s new refugee proclamation also allows refugees to have some access to farmland (FDRE 2019). It would be interesting to investigate whether and how the large IDP and refugee populations in Ethiopia play out and shape the patterns of access to and use of land and labour within host areas.

## The pattern of seasonal migrant labour and dynamics of exploitation

Commodity production by semi-mechanized, export-oriented farmers in the northwestern lowlands of Ethiopia bordering eastern Sudan remains the major destination for seasonal migrant farmworkers every year. Although consolidated and up-to-date data on the number of seasonal migrant farmworkers are hard to find,^1^ over half a million are estimated to work annually on commercial farms in Humera and Metema areas in northwestern Ethiopia and across the border in eastern Sudan. The commercial farms in these lowland borderlands use migrant labour to produce mainly sesame, cotton and sorghum, with sesame one of Ethiopia and Sudan’s major agricultural export commodities.

According to one source, there are more than 400 large-scale commercial farms around Humera, employing over 200,000 seasonal farmworkers during peak seasons (REF 2016c: p. 15; see also Asfaw et al. 2010: p. 62). Additionally, more than 100,000 seasonal migrant labourers are believed to work on the commercial farms around Metema region annually (Asfaw et al. 2010: p. 62). Similarly, following the recent expansion of commercial farms in the Benishangul-Gumuz region, a growing influx of highland seasonal migrant labourers is coming to the area for wage employment (Moreda 2015: pp. 528 − 31). Commercial farms around Metekel, mainly in Dangur and Guba bordering with the Blue Nile state in Sudan, attract tens of thousands of seasonal migrant workers each year. Most of these seasonal wage labourers are landless young men and those with small landholdings unable to provide for their families from such holdings.

There is also significant informal cross-border labour movement of labour. Although seasonal migrant labourers often move between commercial farms located in the northwestern lowlands within Ethiopia, a considerable number also move to Sudan seeking work on the commercial farms there, especially in the neighbouring Gedaref state (REF 2020), where they mainly engage in weeding and harvesting sesame, cotton and sorghum. They migrate to Metema and Humera in April-July, returning to their villages in September. They leave again in November for the harvesting season, returning to their villages between January and February. However, after the harvesting season is over in December on the Ethiopian side, some of the workers move to farms in Gedaref state in Sudan for sorghum harvesting. Some of them stay there for sesame weeding work in June-August, while others return to the farms on the Ethiopian side for the start of the next agricultural season around April/May.

A similar pattern of seasonal labour movement between Ethiopia and Sudan also exists along the Guba corridor in the Benishangul-Gumuz region. A recent report by the Research and Evidence Facility (REF), a study commissioned by the EU Trust Fund, describes this pattern as follows:

There is also seasonal labour migration between Ethiopia and Sudan. The Guba exit in Metekel is dominated by labour migration. Most of the farm workers recruited and employed by investors in commercial farms in Ethiopia migrate irregularly to Sudan when weeding is over in the Ethiopian side, which ends mostly in the middle of September. This season coincides with the ripening of corn farms in the Sudanese side of the border. The Ethiopian workers thus irregularly migrate to Sudan to work in these Sudanese corn farms. …. The workers usually return in April, which means they can work again in commercial farms in the Ethiopian side before irregularly migrating again into Sudan (REF 2016b: p. 25).

A recent news article in the European Union Emergency Trust Fund for Africa (EUTF) reports that some 80,000 Ethiopian seasonal migrant labourers may be crossing the border with Sudan every year to work on commercial farms in the Gedaref state where demand for seasonal labour is usually high (European Commission 2018). Another source gives an even higher estimate of “up to half a million Ethiopian seasonal labour migrants working in agricultural production in Gedaref state on an annual basis” (see ILO 2020a: p. 14). Undoubtedly, the estimates indicate that the extent and impact of such patterns of cross-border seasonal labour migration is significant, and policymakers have already started to draw attention to it. For instance, in 2018, the first workshop on cross-border seasonal labour migration was organized in Khartoum during which government representatives from both countries and representatives of intergovernmental organizations (IGAD, EU and ILO) and civil society discussed how to govern cross-border labour migration (European Commission 2018).

**Fig. 1:**
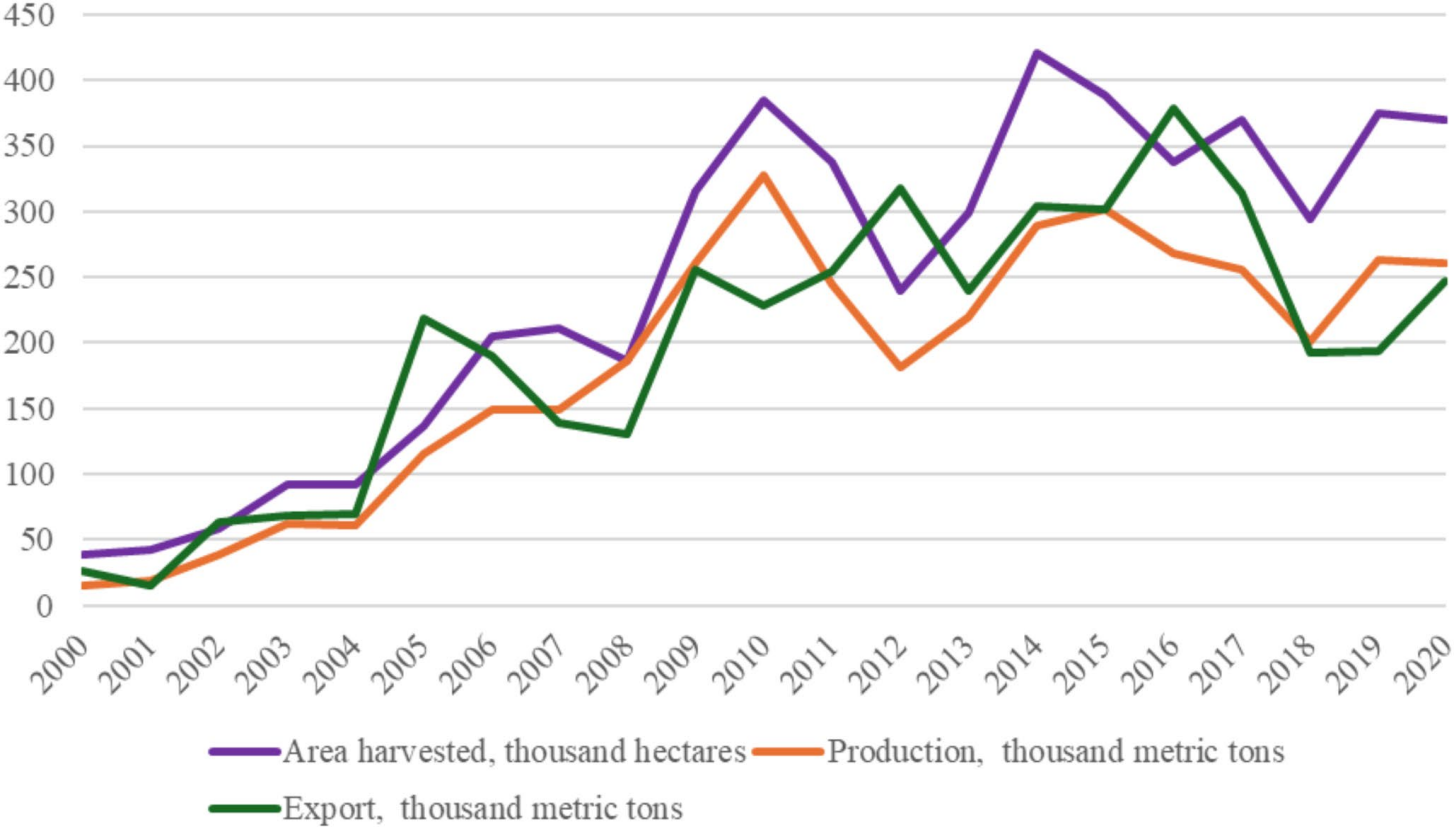
Sesame – area harvested, production and export quantity, Ethiopia. Source: FAOSTAT, https://www.fao.org/faostat/en/#search/sesame [Accessed August 8, 2024]

**Fig. 2:**
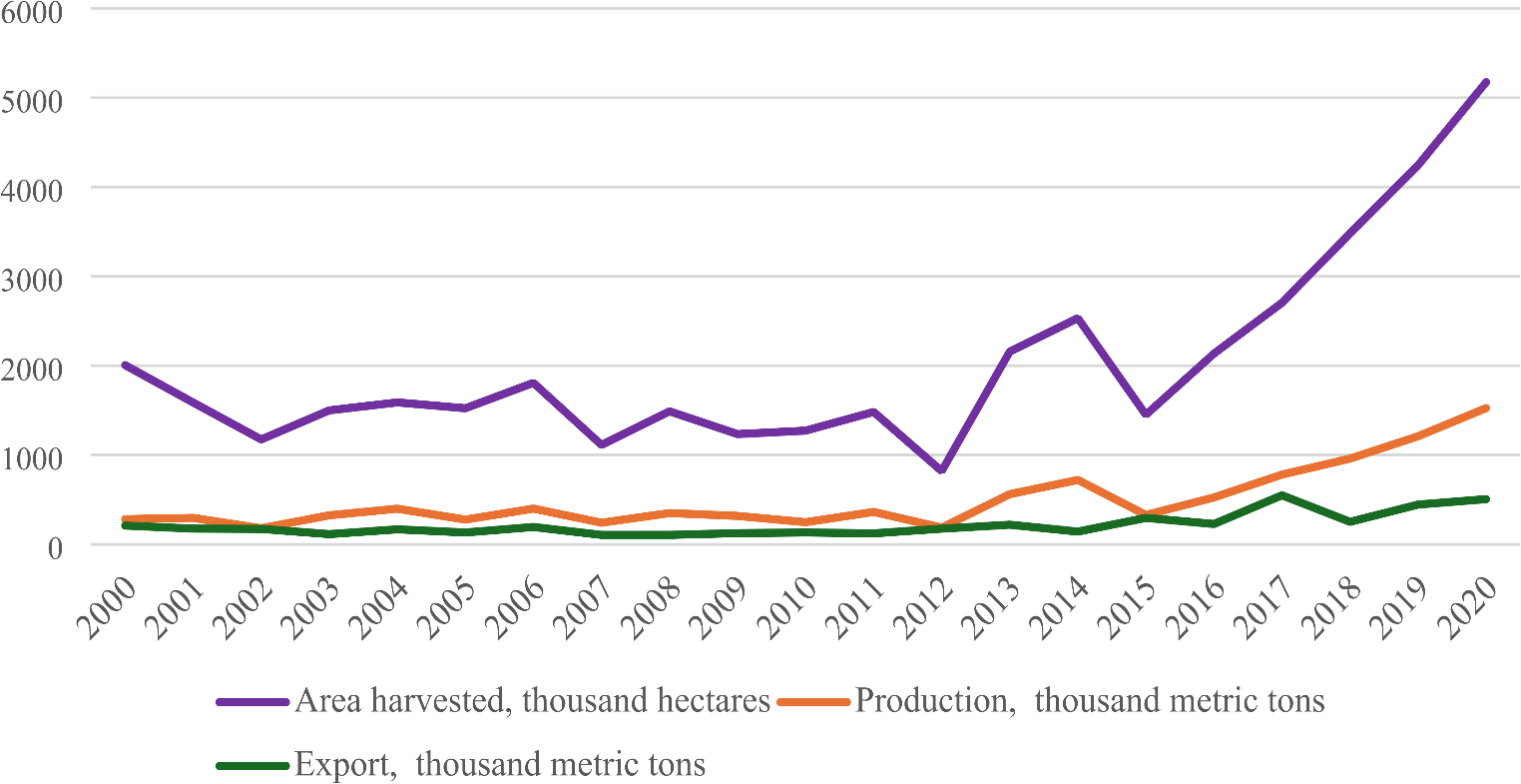
Sesame – area harvested, production and export quantity, Sudan. Source: FAOSTAT, https://www.fao.org/faostat/en/#search/sesame [Accessed August 8, 2024]

The large-scale production of major export commodities, mainly sesame and cotton, on commercial farms on both sides of the Ethiopia-Sudan border are made possible by the availability of vast, cheap, flexible and exploitable seasonal migrant labour. Both Sudan and Ethiopia are currently among the five biggest exporters of sesame globally (FAOSTAT 2024). As data from FAO shows, sesame seed export from both countries has grown over the past several years (Figs. 1 and 2). In 2020, sesame was planted on 369,897 hectares of land in Ethiopia, mainly in the northwestern lowlands, and produced about 260,257 metric tons, of which 247,501 metric tons were exported. The area planted with sesame in Sudan was about 5.2 million hectares, which produced about 1.5 million metric tons, of which 506,056 metric tons was exported. However, sesame yields, especially in Sudan, are very low. Despite this, sesame producers can compete in the export market with other sesame-producing countries with relatively higher yields, with the availability of cheap labour being partly responsible for the profitability of the Sudanese and Ethiopian sesame farmers: “These workers are paid so little that commercial farmers have little incentive to invest in productivity improvements and increased production involves further over-exploitation of both land and labour” (Gallopin et al. 2021: p. 10). The exploitation of workers is deep and pervasive; at times, they are coerced “at the point of a gun” (ibid.).

The origin, scope, causes, dynamics and character of cross-border seasonal labour migration along the Ethiopia-Sudan border is more diverse, complex and fluid than previously understood. While most labour migrants are agricultural workers moving within and between Ethiopia and Sudan, taking advantage of the different farming seasons and labour demand, some seek jobs in the service (restaurants and bars) and other sectors. Some are refugees trying to travel further afield to other countries. The cross-border mobility along the border is a lucrative business that involves brokers, human traffickers and smugglers, and border officials brokering the movement of migrants (Gezahegne 2020). Seasonal migrant labour is characterized by fluid, interlinked and multiple directions of flows within and across sectors and national borders (including rural-rural, rural-urban, urban-rural, agricultural and non-agricultural). While seasonal labour migration in Ethiopia currently constitutes mostly landless young rural labourers, it also constitutes those who continue to have access to small plots of land in their villages but whose land is not deemed sufficient for their social reproduction. Others are urban young people who seldom find their way into the labour market in urban areas. The migrant labour population also includes those affected by violence and ethnic conflict, and climate-induced crises. Others may join the surplus population as a result of expulsion from the land caused by land grabbing. The various processes creating this surplus population mean that the scope and scale of migrant labour will continue to expand as Ethiopia’s working-age population is projected to grow by two million every year over the next decade (World Bank 2019).

Interestingly, seasonal wage labour migrants with access to small plots of land in their places of origin ensure that household members left behind take care of the cultivation of their lands back home. Such land is crucial for a significant part of their households’ subsistence and social reproduction as migrant labourers are usually paid low wages that barely cover their subsistence needs and other costs, such as travel costs (Gallopin et al. 2021). Wages are usually insufficient to cover the living costs of migrants and their families during the rest of the year when they return to their place of origin. This is the case even when the wages in the migrant-receiving areas are higher than rates in their place of origin.

The migrants I interviewed who had just returned to their home villages in Tach Gayint commonly emphasized that the income that they take back home was usually too small to put into any productive activities and is usually used to complement the subsistence needs of their families. There were many cases where migrants returned home sick, most commonly with malaria infections, so the little savings they might have managed to return with were spent on medical treatment. The following case of a 25-year-old married man interviewed in Tach Gayint illustrates this. He has two daughters and has access to a small plot of land. Like many in his village, he had just returned from Humera, where he had worked for two months as a migrant farmworker. His wife stayed at home caring for the family and farmland while he was gone.

I went to Humera in July for agricultural wage work since we could not produce enough food for a year from our small plot of land. I was employed for two months weeding in one of the sesame farms there. I saved some money from my wages during my short stay. Before traveling to Humera, I did the cultivation (field preparation and ploughing) and planting of our land here. And my wife did the weeding with the help of my relatives. I returned in September and did the rest of the activities, such as mowing and harvesting. The wage rate in Humera is higher than in our area. But there are lots of challenges too. I was ill for some time because of malaria and typhoid. I have since gone to health centres two times for treatment. I do not feel okay. It has been three months now since I came back from Humera. I am worried that this malaria may not leave me soon. (18 Jan. 2012, Agatt *kebele*).

During one of my fieldwork visits to Enjit village in Tach Gayint, villagers mourned two migrant youth who died of malaria. One informant explained the case of one of the deceased:

It was yellow fever that took his life. The father is old and weak. His elder son went to the lowlands looking for a job. Malaria caught him there. By the time his younger brother arrived at the hospital, he was already dead. The younger brother came back with empty hands, burying his dead brother there. This is the reason why we were giving condolences to the parents of the deceased. Sadly, the younger brother came back sick with malaria and remained confined in bed. (7 Oct. 2012, Enjit *kebele*).

When migrants come back ill, as in the cases above, the costs for their treatment and care are taken up by their families and socio-cultural community support networks. Thus, the costs of reproducing the migrant labour force for the next season are shouldered by their households, families and communities. Rural communities in Tach Gayint have a long tradition of helping each other in times of difficulty through informal social networks/associations (*Mahiber*):

We have an association called *Misrak* through which we help each other. Last season, three of our association members migrated in search of employment, leaving their families behind. Their families suffered a lot as they did not have anything to sustain them. We contributed money and distributed it to the wives of those members who temporarily migrated, as the wives were left here to take care of the children and the farmland on their own. We also sometimes help them with their farms, including weeding, mowing and harvesting. (14 Oct. 2012, Enjit *kebele*).

Social reproduction in rural life is not limited to the household. “They are always embedded in other social formations that matter for expanded processes of social reproduction and agrarian change, including kinship networks, the community and grassroots groups” (Mezzadri et al. 2024: p. 12). For many male household heads who have access to small parcels of land and seasonally leave their homes, migration is usually a necessary complement to what they can earn on their smallholdings. In such cases, migration decisions involve collective discussions among household members regarding whether to go, who should go and when to go. Crucial to these decisions is ensuring that the land and the family are looked after. The following case of a household head interviewed in Enjit *kebele* shows how migration decisions take place within households, highlighting how critical social reproduction activities are when it comes to how migrant work is organized:

Last season, I decided to go for a few months, hoping to come back with something for my family, but my eldest son insisted that he should be the one to go. Since it was not my first time, I convinced him to stay home to care for his mother and the rest of the family and ensure the land was not left uncultivated, although we don’t usually get much from it. (7 Oct. 2012, Enjit *kebele*).

Another informant, an experienced migrant household head, explained that when he went away from home, his wife strove to generate some income. This included cultivating their land and preparing and selling local drinks. The money she generated was used for household subsistence, whilst the money he brought back from several rounds of migrant wage work was saved.

“The migrant farmworkers must transfer their own familial and community responsibilities to others” (Fraser 2016: p. 114) who stay in the villages. This in effect means that social reproductive capacities are squeezed, especially given that the main migrant-sending areas are often those already struggling with food insecurity and subsistence crises due to land shortages, conflict, drought and environmental degradation.

The families and home communities of the migrants are therefore also affected by seasonal labour migration as they participate in enabling the migration. Thus, capitalist production and accumulation in the borderlands depend not only on the appropriation of value from migrant labour at the production sites but also on social reproductive activities shouldered by migrants’ families and kinship who stay behind to take care of their households. Most of this reproductive work is performed by women. As Fraser aptly puts it, social reproduction is an indispensable condition for sustained capital accumulation.

Non-waged social-reproductive activity is necessary to the existence of waged work, the accumulation of surplus value and the functioning of capitalism. None of those things could exist in the absence of housework, child-rearing, schooling, affective care and a host of other activities which serve to produce new generations of workers and replenish existing ones, as well as to maintain social bonds and shared understandings. (Fraser 2016: p. 102).

In this vein, the social reproductive activities shouldered by the migrants’ households, families and kinship networks form a necessary condition for the patterns of seasonal labour migration that enable workers to migrate and reproduce the next generation of migrants and thus sustain capitalist production and accumulation. In effect, the non-waged/unpaid family members, particularly women, subsidize the waged male labour and is, in turn, a subsidy to capitalist production and accumulation (see Mezzadri 2021: p. 1188; see also Shivji 2017; Benya 2015; Yeni 2024). Key classic studies about seasonal labour migration have long highlighted such dynamic interpenetration between production and social reproduction realms and activities and why the two cannot be separated. For example, Meillassoux (1972) discussed how capitalism utilizes agricultural communities to partly provide for the reproduction of labour power. Drawing from observations in Southern Africa, Meillassoux noted that by utilizing migrant wage labour, gold mining schemes were able to dump the cost of reproducing migrant labour onto their families and communities in the migrants’ areas of origin (see Benya 2015 for a recent insight). Similarly, Burawoy (1976) showed how employers externalized the cost of reproduction of migrant labour by using migrant farmworkers in California and migrant mine workers in South Africa (see also Cousins et al. 2018). Based on insights across India, Mezzadri (2021) outlines how informalized labour processes and regimes enable the externalization of a significant portion of costs for the social reproduction of labour, for instance, when employers pass it onto workers’ families and communities in migrant workers’ home areas. A recent study by Shah and Lerche precisely captures such dynamics, demonstrating the intertwinement of productive and social reproductive activities in seasonal labour migration in India, “the costs of which are borne by the worker and their kin” (2020: p. 722).

The seasonal migrant farmworkers themselves face several direct challenges and exploitative conditions in the commodity-producing borderlands. In addition to exposure to malaria and other health risks, the seasonal migrants interviewed in Tach Gayint mentioned the harsh daily labour and the long working hours with no shelter in the baking hot temperatures of the lowlands. The migrants explained that with group contractual arrangements, they usually team up with seven or eight others based on their areas of origin and/ or ethnicity to weed, mow or harvest a specific size of crop field. Each group competes (often violently) with other groups for the available jobs. Once contracted, the group stays on the farm day and night with only minimal accommodation until the agreed work is completed. Employers do not usually provide them with accommodation, which exposes them to harsh weather conditions. A young migrant described the challenges as follows:

We suffer from hunger and easily get sick. The water we drink is not good either. We get it from open barrels filled and left in the open air, exposed to the sun the whole day in that hot climate. It is not difficult to imagine how bad it could taste and affect our health. (12 Jan. 2013, Enjit *kebele*).

Another form of grouping is between early migrants to the areas who have made the destinations their home. Locally identified as *Sallug*, this group includes not only experienced seasonal migrants but also people who moved and settled in the areas through government resettlement programs. They are in conflict with new first-time migrants called *Goffers*. The *Sallug* fear that the massive entrance of the *Goffer* to the lowlands will take away employment opportunities. They also fear that wage rates may decrease due to a new batch of young men flocking seasonally to the area. This has led to attacks and abuse of the *Goffers*.

Furthermore, armed groups and militias rooming the borderlands also informally tax migrant agricultural workers, further squeezing them of their hard-earned small wages (Gallopin et al. 2021). Yet despite the challenges and poor working conditions, the borderlands remain key destinations for seasonal migrant farmworkers, a testament to their resilience and the crucial role in capitalist production in the area.

## Conclusions

Agricultural commodity production and migrant labour are intimately linked in a very central way. As discussed in this paper, over half a million Ethiopian seasonal agricultural labourers, predominantly from the Amhara and Tigray regions, move across the lowlands to work on farms on both sides of the border between Ethiopia and Sudan. Seasonal agricultural wage work is indeed a crucial livelihood strategy for these migrant farmworkers who find it challenging to make a living from their small plots of land and for those faced with diminished prospects of accessing land and limited employment opportunities in their home villages. However, to frame migrant labourers as mere beneficiaries of employment opportunities is to lose sight of their crucial role in shaping the political economy of commodity production in the borderlands and the varied domains of exploitation they face. This paper has highlighted why and how cross-border seasonal migrant labour should be taken as an inextricable element of capitalist commodity production in the Ethiopia-Sudan borderlands. Its enormous scale alone is indicative of its significance in commodity production.

Due to large-scale sesame production in these borderlands, Ethiopia and Sudan are among the five biggest exporters globally. This is connected to, and rooted in, the availability of vast, cheap, flexible and exploited migrant agricultural labour. The importance of such migrant labour to commodity production is conditioned and shaped by the fact that most of these migrant labourers are ‘rurally rooted’; they maintain their small plots of land, and their families in their home villages providing much-needed safety nets, thus ensuring that the cost of migrant labour is kept below its reproduction cost. This dynamic ensures that the production of sesame and other commodities remains lucrative for commercial farmers. The result is that by “putting more pressure on the already resource-stretched households and communities” (Benya 2015: p. 557), “social reproductive capacities are further squeezed” (Fraser 2016: p. 114). This echoes the notion that social reproduction is not something that exists outside the processes of capitalist production and accumulation (Mezzadri 2021: p. 1187).

This paper has highlighted why the over-emphasis on the dynamics of labour exploitation in migrant work areas, including poor and harsh working conditions as well as the low wage-centric understanding of exploitation, is incomplete. This over-emphasis further obscures the fact that the exploitation of migrant labour manifests in multiple forms, occurring simultaneously in the labour-destination areas and in their place of origin. Here their kin, particularly women, shoulder the household subsistence and labour reproduction responsibilities, effectively subsidizing the commodity production and accumulation in the labour-destination areas. This underscores the importance of recentring the analysis of migrant labour relations and processes and the complex forms of exploitation surrounding them. These must necessarily be understood by focusing on both migrant workers’ destination and their place of origin and on production and social reproduction. This will allow for a better understanding of the domains of seasonal migrant labour exploitation in the struggles for labour justice and to inform social policies.

## Footnotes

1 This is mainly because most migrate irregularly and due to the informality of the type of work and nature of employment conditions to which most are subjected.
